# A New Soldier-Producing Aphid Species, *Pseudoregma baenzigeri,* sp. nov., from Northern Thailand

**DOI:** 10.1673/031.007.3801

**Published:** 2007-06-11

**Authors:** Shigeyuki Aoki, Utako Kurosu, Warunee Sirikajornjaru

**Affiliations:** ^1^ Faculty of Economics, Rissho University, Osaki 4-2-16, Tokyo, 141-8602 Japan; ^2^ Faculty of Economics, Chuo University, 742-1 Higashinakano, Hachioji, Tokyo 192-0393, Japan; ^3^ Department of Plant Protection, Faculty of Agricultural Production, Maejo University, Chiang Mai 50290, Thailand

**Keywords:** Aphididae, bamboo, bionomics, Cerataphidini, Hormaphidinae, *Dendrocalamus*

## Abstract

*Pseudoregma baenzigeri*, sp. nov., is described from northern Thailand. This species forms dense, huge colonies on shoots of the bamboo *Dendrocalamus* sp., and produces many first-instar, pseudoscorpion-like soldiers. Alate sexuparae were found from the end of September to mid October. Two syrphids, *Eupeodes* sp. A (allied to *E. confrater*) and *Dideoides chrysotoxoides,* and the pyralid *Dipha aphidivora* were recorded as predators of P. *baenzigeri*. The aphids were also likely to be eaten by some rodents. The apterous adult, nymphs, soldier and alate sexupara of P. *baenzigeri* can be distinguished from those of the other congeners by the longer, conical ultimate rostral segment. A tentative key to the species of *Pseudoregma* living on bamboo is provided.

## Introduction

The aphid genus *Pseudoregma* (Hormaphidinae, Cerataphidini) comprises about a dozen species, all of which, including one widely distributed species (*P. panicola* (Takahashi)), have been recorded from the tropical and/or subtropical regions of Southeast Asia (Blackman & Eastop 1994). Four of them are known to induce banana-bunch shaped galls on trees of the genus *Styrax*, their primary host (Aoki & Kurosu 1991, 1992; Kurosu & Aoki 2001; Aoki et al. 2002), and the other species are also supposed to be associated with *Styrax*. Their secondary hosts are monocotyledons such as bamboo, grass and ginger (Blackman & Eastop 1994). All but one unnamed species (Stern et al. 1997) produce morphologically distinct “pseudoscorpion-like” soldiers in their exposed colonies on the secondary host. The soldiers are of the first instar, and have greatly thickened forelegs and a pair of sharp frontal horns. They tightly clasp a predator with their forelegs and pierce it with their frontal horns (Aoki & Miyazaki 1978; Aoki et al. 1981). Because of the production of the bizarre soldiers, the genus has recently attracted attention from researchers and has been studied from various aspects, such as the allometry (Stern et al. 1996), longevity (Tanaka & Itô 1994), physiology (Arakaki 1992; Arakaki & Hattori 1998) and embryology of soldiers, (Ijichi et al. 2004, 2005), molecular phylogeny (Fukatsu et al. 2001), interaction with predators and/or ants (Ôhara 1985a, b; Schütze & Maschwitz 1991; Shibao 1998; Shingleton & Foster 2000, 2001), and factors affecting the proportion of soldiers (Sunose et al. 1991; Shibao 1999; Aoki & Kurosu 2003, 2004; Aoki & Imai 2005).

Through the courtesy of Dr. Hans Bänziger, we had an opportunity to examine samples of a *Pseudoregma* species collected from bamboo (*Dendrocalamus* sp.) on hills near Mae Sai, Mae Sai District, Chiang Rai Province, northern Thailand. The samples contained many apterous adults, nymphs, pseudoscorpion-like soldiers and several alates. Having examined the morphology of these morphs, we came to the conclusion that the species is new to science. Below we describe the species as a new species, present some biological information offered by Hans Bänziger, and provide a tentative key to the species of *Pseudoregma* living on bamboo.

## Materials and Methods

The aphid specimens used for the description were collected by Hans Bänziger from colonies on *Dendrocalamus* sp. found near Mae Sai, Tailand in 1994, 2000 and 2001, and were preserved in 70–80% ethanol. For slide preparation, aphids were cleared in heated 10% KOH solution. These aphids were stained with either Evans' blue or acid fuchsin, dehydrated in a mixture of glacial acetic acid and methyl salicylate for a few days, and mounted in balsam via a mixture of xylol/phenol and pure xylol. Some were mounted without being stained. In the following description, unless the sample size is indicated in parentheses, measurements for the normal first-instar nymph, soldier and apterous adult were based on 10 specimens collected on 13 October 2001, and those for the alate sexupara on seven specimens collected on 29 September 1994 and on 7 and 14 October 2001. We also examined a total of 77 slide-mounted soldiers to determine whether they had the second instar cuticle developing inside.

## Description

### Pseudoregma baenzigeri sp. nov.

#### Normal 1st-instar nymph (Figures 1 & 16)

Body (excluding horns) 0.93–1.21 (mean 1.09) mm. Cephalothorax, most of the mesotergum, sides of metatergum and cauda sclerotized. The rest of metatergum and first to seventh abdominal tergites membranous, but bases of spinal and marginal setae sclerotized to form sclerotic patches. Eighth abdominal tergite with a wide sclerotic plate. Tergites with numerous wart-like granules, which are remarkable on sclerotic part. Wax plate composed of one to several more or less distinctly demarcated cells, occurring on sclerotic part. Head with a pleural pair of wax plates dorsally between eyes; all thoracic tergites and first to sixth abdominal tergites each with marginal and spinal pairs of wax plates, but spinal plates often reduced in size or disappearing, in particular on posterior abdominal segments; seventh abdominal tergite with a marginal pair of wax plates; eighth abdominal tergite with a single wax plate composed of a transverse row of approximately 10–16 cells. Frontal horns sharp, a little longer than third antennal segment, with a number of minute setae that are up to 0.002 mm long. Head with a few pairs of long setae along the anterior margin above frontal horns, some of which reach apical four-fifth of the horns. Antenna four-segmented; fourth segment 0.106–0.140 (0.121) mm long, weakly imbricate, with five apical setae and two basal setae, the longer one of which is 0.052–0.060 (0.056) mm (n = 8); first, second and third segments 0.048–0.064 (0.057) mm, 0.040–0.066 (0.049) mm and 0.088–0.126 (0.105) mm long, and with two, two and from six to 11 setae, respectively; primary rhinarium on third segment distinctly rimmed, composed of a single spherical rhinarium, 0.014–0.018 (0.016) mm in axial length; primary rhinarium on fourth segment also distinctly rimmed, composed of an ellipsoidal rhinarium and a few smaller accessory rhinaria, 0.022–0.030 (0.026) mm in axial length. Rostrum reaching hind coxae; ultimate segment (Figure 16) conical, 0.104–0.130 (0.119) mm long, without secondary setae. Foreleg not thickened, with normal claws; femorotrochanter 0.31–0.38 (0.34) mm long (n = 9), 0.070–0.084 (0.078) mm wide (n = 9). Hind femorotrochanter 0.32–0.42 (0.37) mm long; second segment of hind tarsus 0.136–0.178 (0.155) mm long. Dorsoapical setae on tarsus long and capitate, but inner one on foreleg usually reduced; empodial setae spatulate, extending beyond the apices of claws. Cornicle absent. First to sixth abdominal tergites each with more than six setae; the marginal setae very long, 0.094–0.120 (0.106) mm on third tergite (n = 9); third tergite with eight to 12 (10.1) setae; seventh and eighth tergites with six to eight (6.8) and four or five (4.1) setae, respectively. Cauda with two or three (2.2) setae. Anal plate with four to six (4.7) setae (n = 9).

**Figures 1–3.**
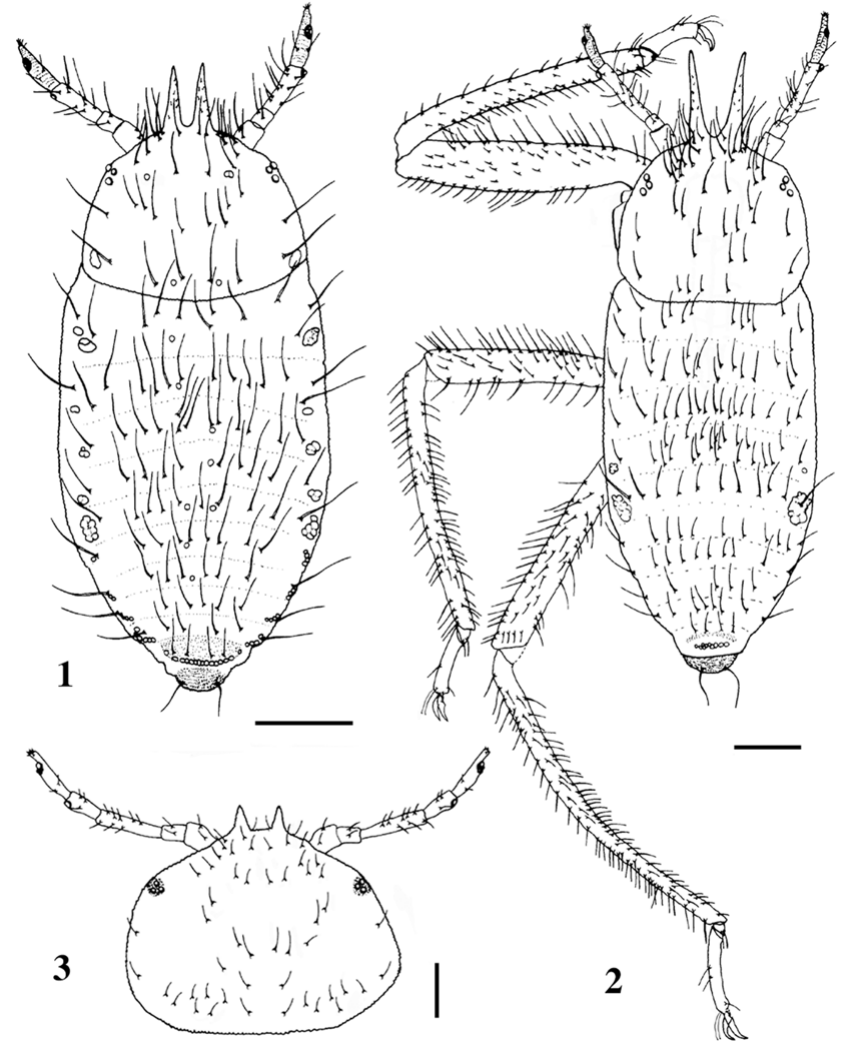
*Pseudoregma baenzigeri*. 1 Body of normal ist-instar nymph, 2 soldier, 3 head of apterous adult. Scale lines: 0.1 mm.

**Figures 4–8.**
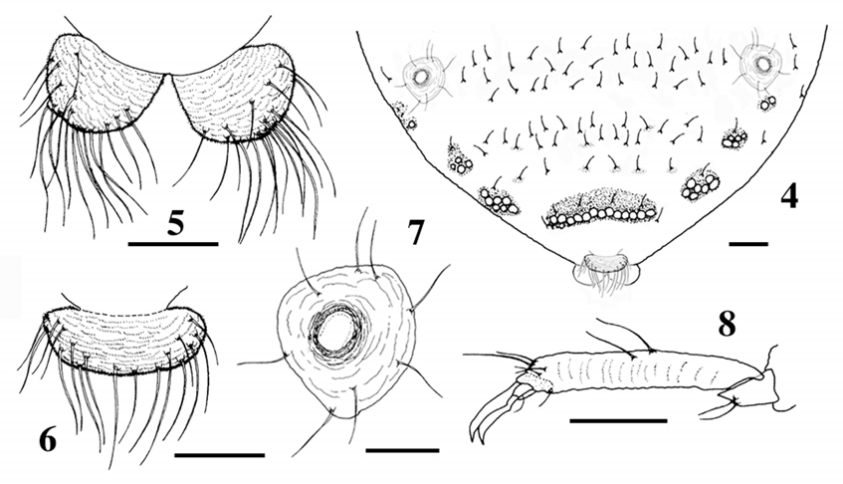
Apterous adult of *Pseudoregma baenzigeri*. 4 Posterior abdominal tergites, 5 anal plate, 6 cauda, 7 cornicle, 8 hind tarsus. Scale lines: 0.1 mm for 4, and 0.05 mm for 5–8.

### Soldier (Figure 2)

Much larger than normal first-instar nymph, but variable in size. Anterior part (forelegs and horns, in particular) enlarged and strongly sclerotized. Body (excluding horns) 1.56–1.80 (1.71) mm. Cephalothorax, meso- and meta-thoracic tergites sclerotized. Anterior abdominal tergites extensively sclerotized; posterior tergites with sclerotic patches. Tergites with numerous wart-like granules, as in the normal first-instar nymph. Wax plates reduced; no wax plates on head, thorax or first abdominal tergite; second abdominal tergite at times with marginal wax plates; third abdominal tergite with a pair of large, marginal wax plates; eighth abdominal tergite with several oval wax gland cells along the posterior margin. Frontal horns long, acute at apex, clearly longer than third antennal segment, with several minute setae. Antenna four-segmented; third segment 0.192–0.260 (0.222) mm long with 12–16 (14.5) setae.

Ultimate rostral segment conical, 0.142–0.152 (0.146) mm long, without secondary setae. Foreleg greatly thickened, with large, strongly curved claws; femorotrochanter 0.69–0.85 (0.78) mm long, 0.134–0.176 (0.151) mm wide. Hind femorotrochanter 0.71–0.85 (0.78) mm long; second segment of hind tarsus 0.244–0.284 (0.267) mm long. Cornicle absent. First to sixth abdominal tergites each with more than six setae (but sometimes six on sixth tergite); the marginal setae very long, 0.080–0.102 (0.091) mm on third tergite (n = 8); third tergite with 10–13 (11.2) setae; seventh and eighth tergites with six to eight (7.0) and four to six (5.7) setae, respectively. Cauda with two setae. Anal plate with four to seven (5.4) setae.

None out of 77 soldiers had the second instar cuticle developing inside, indicating that the soldiers are sterile.

### Apterous adult (Figures 3–8 & 12)

Color in life grayish green. Body (excluding horns) 2.8–3.6 (3.2) mm. Cephalothorax, most part of mesotergum, sides of metatergum and cauda sclerotized. First to seventh abdominal tergites each with a pair of sclerotic plates marginally and often with smaller sclerites between them; eighth tergite occupied by a large sclerotic plate. Tergites with numerous pustules, which are distinct on sclerotic part. Head (Figure 3) with a pair of frontal horns, which are roughly as long as first antennal segment, curved upward and with a few short setae. Antenna four- or five-segmented; the apical segment weakly imbricate, 0.172–0.204 (0.190) mm long, with two setae on basal half and five short setae at apex, with a large primary rhinarium, which is 0.034–0.038 (0.037) mm in axial length and consists of an ellipsoidal rhinarium and a few smaller accessory rhinaria; primary rhinarium on penultimate segment 0.018–0.022 (0.021) mm in axial length. Ultimate rostral segment (Figure 12) conical, 0.150–0.162 (0.156) mm long (n = 6), without secondary setae. Hind femorotrochanter 0.95–1.13 (1.07) mm long. Tarsi (Figure 8) two-segmented; first segment with a pair of setae, which are 0.034–0.046 (0.038) mm long on hind leg, and with two shorter sense pegs on fore and mid legs; second segment 0.212–0.252 (0.233) mm long, with the dorsoapical setae thin and not capitate; empodial setae thin. Wax plate consisting of round facets (see Figure 4); a marginal pair on first to seventh abdominal tergites, but usually reduced in size and often disappearing on anterior (first to fourth) segments; a small spinal wax plate occurring on sixth abdominal tergite of one specimen; eighth abdominal tergite with a large wax plate consisting of 13–20 (15.2) facets (n = 9), and with eight to 10 (8.6) setae (n = 7). Cauda (Figure 6) constricted at base, 0.164–0.192 (0.179) mm wide, with 17–20 (18.3) setae (n = 6). Anal plate (Figure 5) bilobed, with a total of 33–36 (33.8) setae (n = 6). Genital plate with four to seven (5.3) setae on anterior half and with 24–29 (27.3) setae on posterior half, mainly along the posterior margin (n = 4). Cornicle (Figure 7) ring-like, 0.076–0.086 (0.083) mm in outer diameter (n = 9), on weakly sclerotic cone, encircled by seven to 11 (8.8) setae (n = 8).

**Figures 9–11.**
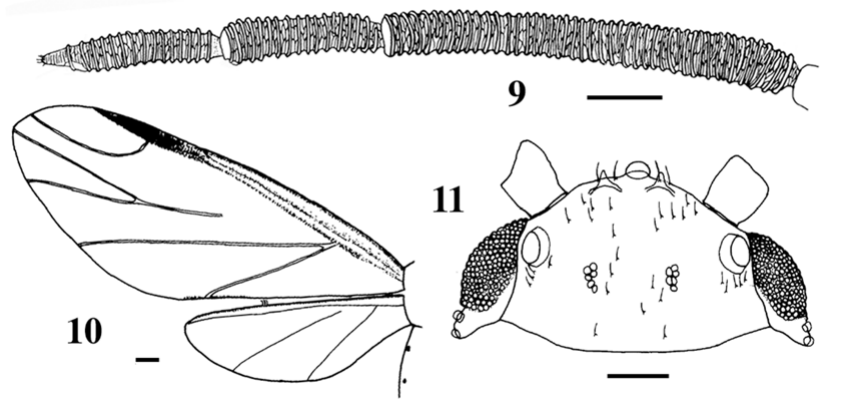
Alate sexupara of *Pseudoregma baenzigeri*. 9 Antenna, 10 wings, 11 head. Scale lines: 0.1 mm for 10, and 0.05 mm for 9 and 11.

### Alate sexupara (Figures 9–11)

Body (excluding horns) 2.2–2.6 (2.5) mm long. Head (Figure 11) dark brown, bearing a pair of short, yet distinct frontal horns, dorsally with a number of short setae which are 0.012–0.024 (0.018) mm long. Frontal horn approximately 0.030–0.036 (0.032) mm long, with a number of minute setae that are less than 0.004 mm long. Antenna (Figure 9) five-segmented, longer than fore tibia; third segment thicker than fore tibia, 0.54–0.60 (0.57) mm long, longer than fourth and fifth segments together; fourth segment 0.18–0.28 (0.21) mm long, a little shorter than fifth except for one specimen; fifth segment 0.20–0.25 (0.22) mm long; processus terminalis conical, with five short setae at apex. Secondary rhinaria ring-like, encircling more than half the segment; third, fourth and fifth segments with 41–47 (44.4), 13–20 (16.7) and 12–19 (14.7) ring-like rhinaria, respectively; apical two or three and apical one to four rhinaria on fourth and fifth segments, respectively, united with the primary rhinarium. Primary rhinaria irregular in shape, on fifth segment ciliated, 0.026–0.034 (0.030) mm in axial length, on fourth segment not ciliated, 0.014–0.024 (0.020) mm in axial length. Clypeus ventrally with a number of distinct, wart-like projections. Ultimate rostral segment conical, 0.136–0.140 (0.139) mm long, without secondary setae. Forewing with media once branched, and with cubital branches arising from a common stem; hind wing with two oblique veins (Figure 10). Hind femorotrochanter 0.69–0.73 (0.71) mm long. Tarsi almost smooth; first segment with a pair of setae which are 0.024–0.030 (0.027) mm long on hind leg, and on fore and mid-legs with two shorter sense pegs between the setae; second segment 0.168–0.192 (0.183) mm long on hind tarsus, with a pair of setae mid-dorsally (and sometimes additional one mid-ventrally on hind leg) and three pairs of setae apically, all of which are short and pointed; empodial setae thin, reaching near the apices of claws. Abdomen weakly and extensively sclerotized on seventh and eighth tergites; eighth tergite with nine to 13 (10.6) setae. Cornicle ring-like, 0.020–0.034 (0.027) mm in outer diameter, encircled at a distance by four to seven (5.3) setae (n = 4). Cauda 0.082–0.116 (0.097) mm wide, with 14–21 (17.4) setae. Anal plate bilobed, with a total of 24–26 (25.3) setae. Genital plate with 25–39 (31.3) setae (n = 6), of which 17–27 (22.7) are located along the posterior margin (n = 6).

**Figures 12–19.**
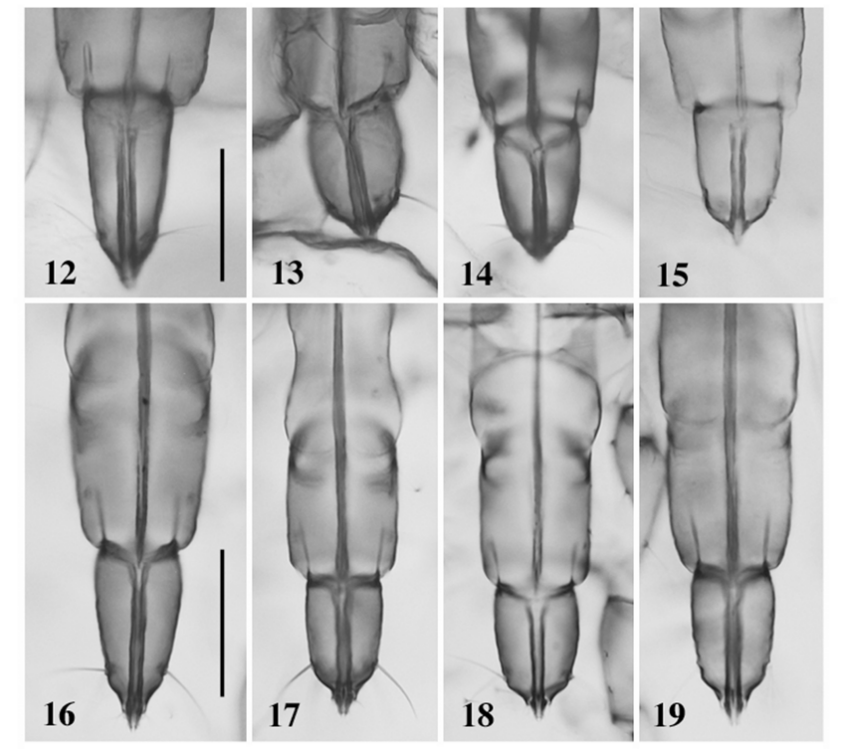
Apical rostral segments of *Pseudoregma* spp. 12–15 Apterous adult, 16–19 normal first-instar nymph: 12 & 16 *P. baenzigeri*, 13 & 17 *P. alexanderi*, 14 & 18 *P. carolinensis*, 15 & 19 *P. bambucicola*. Scale lines: 0.1 mm; the upper line for 12–15 and the lower for 16–19.

### Type materials

Holotype. One apterous viviparous female, hills near Mae Sai, Mae Sai District, Chiang Rai, Thailand, on *Dendrocalamus* sp., 13 October 2001 (Hans Bänziger leg.).

Paratypes. 68 slides including 51 alates, 40 apterous adults, 67 soldiers and 81 nymphs, hills near Mae Sai, Mae Sai District, Chiang Rai, Thailand, on *Dendrocalamus* sp., 29 September 1994, 1 October 2000, 6–13 October and 25 November 2001 (Hans Bänziger leg.).

The holotype and paratypes are deposited in the collection of Maejo Insect Museum, Maejo University, Chiang Mai, Thailand. Some paratypes are also deposited in the collections of Department of Entomology, Faculty of Agriculture, Chiang Mai University (Chiang Mai), Systematic Entomology, Hokkaido University (Sapporo), Department of Zoology, National Science Museum (Tokyo) and Natural History Museum (London).

## Taxonomic remarks

There have been known eight named species of *Pseudoregma* from bamboo (Takahashi 1935, 1950; Aoki et al. 1981; Noordam 1991; Fukatsu et al. 2001). They are *P. alexanderi* (Takahashi), *P. bambucicola* (Takahashi), *P. carolinensis* (Takahashi), *P. dendrocalami* (Takahashi), *P. gombakana* (Takahashi), *P. koshunensis* (Takahashi), *P. montana* (van der Goot) and *P. pendleburyi* (Takahashi). Another unnamed species is known from *Schizostachyum zollingeri* in the Malay Peninsula (Stern et al. 1997). *Pseudoregma baenzigeri* is peculiar in that the apterous adult, alate, normal first instar nymph and soldier all have the ultimate rostral segment which is conical and longer than those of the other species (Figures 12–19), and can be easily distinguished from them. A tentative key, based both on the apterous adult and first-instar nymph, to the species of *Pseudoregma* on bamboo is provided below:

**Table:**
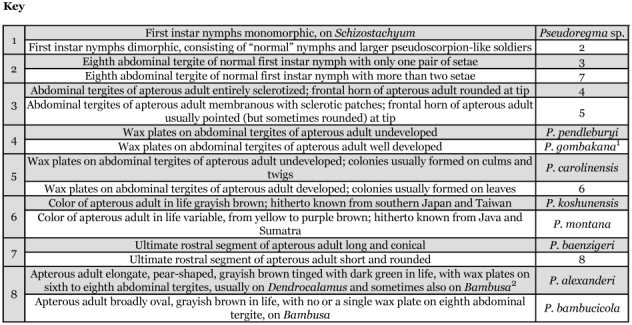


The above key does not include *Pseudoregma dendrocalami*, because no authentic specimen of the species was available. The original description of *P. dendrocalami* by Takahashi (1935) was based on specimens collected by J. C. van der Meer Mohr in Medan, Sumatra. According to Takahashi (1935), the apterous adult of *P. dendrocalami* has “4 dark grayish green broad longitudinal dorsal stripes running from the head to [the middle of] the abdomen.” Except for the color pattern, the apterous adult resembles that of *P. carolinensis*. It should also be mentioned that *P. pendleburyi* and *P. gombakana* might be the same species (Blackman & Eastop 1994), and that the distinction between *P. koshunensis* and *P. montana* remains to be clarified.

## Biology

The following account of the bionomics is based on observations by Dr. Hans Bänziger on hills near Mae Sai from 1994 to 2005.

### Hostplant, season and habitat

*Pseudoregma baenzigeri* forms dense colonies on shoots of a tall bamboo, *Dendrocalamus* sp. Colonies of this aphid were found from September to November, but in 2001 until near the end of December. One to a maximum of four bamboo clumps in an area of some 50 x 50 meters were infested with the aphids, while no infested bamboo was found over the remainder of an estimated 2 km long pathless forest stretch surveyed from 1994 to 2005. In 1997, 2002, 2004 and 2005, no infestation was detected in the surveyed area, which was, however, only a tiny fraction of the forest that remained.

### Infestation

Shoots colonized by aphids could attain approximately 8 m height before the growth ceased. Some shoots were infested with the aphids more or less completely throughout the length, while others only at one or several sections at various heights. On young shoots the sheaths, and on older shoots the terminal part of the culm, were often densely occupied by the aphids. Aphids were generally very densely packed, giving rise to huge populations: about 10 adults or 120 first instar nymphs were counted per square cm, equivalent to 28,000 adults or 340,000 first instar nymphs per meter of a bamboo of 9 cm diameter. The total number of aphids may have exceeded one million on a shoot found on 7 November 2001, which was approximately 7 m in height and nearly completely covered with the aphids from the basal two meters to the top. Such huge populations may have important consequences for the food web system. Ants of *Pheidologeton trechideros* Zhou et Zheng, *Dolichoderus thoradcus* (Smith) and *Myrmicaria brunnea* Saunders were attending the colonies, sometimes under shelters covering part of the colonies. Honeydew falling from the colonies formed a broad dark band on the forest litter. The honeydew was sucked by many insects: blow flies such as *Catapicephala kurahashii* Tumrasvin et Kano and *Lucilia porphyrina* (Walker), various bees and butterflies.

### Insect predators

Despite the production of soldiers, the aphids themselves were eaten by some predators. *Eupeodes* sp. A was the commonest predator. This syrphid is closely related to *E. confrater* (Wiedemann), which is known to prey on *Pseudoregma bambucicola* in southern Japan (Ôhara 1985b; Shibao 1998), but it is a distinct, undescribed species (Nigel Wyatt, personal communication to Hans Bänziger). Third instar larvae of *Eupeodes* sp. A moved through *P. baenzigeri* aggregations without being attacked by soldiers, as those of *E. confrater* do through a colony of *P. bambucicola* (Ôhara 1985b). The syrphid larvae were covered with their preys' wax and it was not easy to detect them hidden in the aphid colonies. In one case (observed on 14 October 2001), a soldier of *P. baenzigeri* crawled onto a larva of the syrphid without showing any sign of attacking it. When the tips of a pair of forceps were put close to the syrphid larva, the soldier immediately attempted to attack the forceps and continued to chase the forceps while walking on the larva as the forceps were moved back and forth over the larva. It seemed as if the soldier intended to protect the syrphid larva, which was perceived as part of the aphid colony. Also, eggs of *Eupeodes* sp. A were often found laid on spider webs near colonies of *P. baenzigeri* (e.g. on 26 October 1994). Direct observations on oviposition by females of this syrphid on nearby spider webs were not made for *P. baenzigeri* but for *P. carolinensis* (on the campus of Chiang Mai University, Chiang Mai, on 4 and 6 November 1994). Such ovipositing behavior is known for *Eupeodes confrater,* and interpreted as an adaptation against attack on the syrphid's eggs by aphid soldiers (Ôhara 1985a; see also Moffett 1989). There was another syrphid species that may be a specialized predator of this and other species of *Pseudoregma.* A female hoverfly of *Dideoides chrysotoxoides* (Curran) laid a dense clutch of more than 120 eggs a few cm from a colony of *P. baenzigeri* (on 19 September 1994). Soldiers did not seem to recognize them as enemy eggs, and no attack on the eggs was observed. It is noteworthy that two congeners of the syrphid, *Dideoides coquilletti* van der Goot and *D. latus* (Coquillett) prey on *Colophina* spp. (Ôhara 1984; Aoki & Kurosu 1999) and *Ceratovacuna* spp. (Aoki & Kurosu 1987), respectively, both of which produce soldiers or defensive nymphs (Aoki 1977; Arakaki 1989). A third hoverfly observed was *Episyrphus alternans* (Macquart), which laid eggs singly on the margin of bamboo sheaths, about 20 cm distant from a colony of *P. baenzigeri* on 7 October 2001, but checks six days later yielded no larvae of this hoverfly, suggesting that they had been disposed of by soldiers. Larvae of *E. alternans* are known to prey on *Aphis gossypii* Glover, one of the commonest aphids in Thailand (Bänziger 1980) and seem not to be adapted to the defensive strategies of *Pseudoregma*. Incidentally, this syrphid is the main pollinator of the orchid *Paphiopedilum callosum*, which tricks the hoverfly into ovipositing onto the flower by a scent mimicking an aphid colony (Bänziger 2002). Carnivorous larvae of the pyralid moth *Dipha aphidivora* (Meyrick), living in silken shelters (Arakaki & Yoshiyasu 1988), were also found from colonies of *P. baenzigeri*.

### Predation by rodents

Aphids of *P. baenzigeri* are likely to be eaten by some rodents. It has been repeatedly observed that aphids were obliterated along two parallel lines from an otherwise homogeneously dense colony, which were evidently marked with the incisor teeth by a rodent harvesting the aphids. Indeed, a squirrel, *Callosciurus erythraeus* (Pallas), was seen to eat aphids of the congener *P. carolinensis* on a bamboo branch near the foot of Doi Suthep, Chiang Mai (on 1 January 2006).

### Appearance of alates

Alates of *P. baenzigeri* appeared from the end of September to mid October. All alate specimens we examined contained hornless, sexual embryos, indicating that they were sexuparae that would fly to the primary host, *Styrax* trees. The primary-host generation is yet unknown.

## Notes

(1) It remains to be determined whether the first instar nymph of *P. gombakana* has only one pair of setae on the eighth abdominal tergite.
(2) We found small colonies of *P. alexanderi* on *Bambusa oldhami* and *B. vulgaris* at Sun Moon Lake, Taiwan, on 16 December 1992 and 24 April 2005, respectively.

### Editor's Note

Paper copies of this article will be deposited in the following libraries. Senckenberg Library, Frankfurt Germany; National Museum of Natural History, Paris, France; Field Museum of Natural History, Chicago, Illinois USA; the University of Wisconsin, Madison, USA; the University of Arizona, Tucson, Arizona USA; Smithsonian Institution Libraries, Washington D.C. U.S.A.; The Linnean Society, London, England.
